# Sequelae and Comorbidities of COVID-19 Manifestations on the Cardiac and the Vascular Systems

**DOI:** 10.3389/fphys.2021.748972

**Published:** 2022-01-14

**Authors:** Yashvardhan Batta, Cody King, John Johnson, Natasha Haddad, Myriam Boueri, Georges Haddad

**Affiliations:** ^1^Department Physiology and Biophysics, College of Medicine, Howard University, Washington, DC, United States; ^2^Lebanese American University, Beirut, Lebanon

**Keywords:** COVID-19, SARS-CoV-2, hypertension, neuropathy, cardiac arrhythmias, hypercoagulation, ischemic heart disease, aging

## Abstract

COVID-19 patients with pre-existing cardiovascular conditions are at greater risk of severe illness due to the SARS-CoV-2 (severe acute respiratory syndrome coronavirus 2) virus. This review evaluates the highest risk factors for these patients, not limited to pre-existing hypertension, cardiac arrhythmias, hypercoagulation, ischemic heart disease, and a history of underlying heart conditions. SARS-CoV-2 may also precipitate *de novo* cardiac complications. The interplay between existing cardiac conditions and *de novo* cardiac complications is the focus of this review. In particular, SARS-CoV-2 patients present with hypercoagulation conditions, cardiac arrhythmias, as significant complications. Also, cardiac arrhythmias are another well-known cardiovascular-related complication seen in COVID-19 infections and merit discussion in this review. Amid the pandemic, myocardial infarction (MI) has been reported to a high degree in SARS-CoV-2 patients. Currently, the specific causative mechanism of the increased incidence of MI is unclear. However, studies suggest several links to high angiotensin-converting enzyme 2 (ACE2) expression in myocardial and endothelial cells, systemic hyper-inflammation, an imbalance between myocardial oxygen supply and demand, and loss of ACE2-mediated cardio-protection. Furthermore, hypertension and SARS-CoV-2 infection patients’ prognosis has shown mixed results across current studies. For this reason, an in-depth analysis of the interactions between SARS-CoV2 and the ACE2 cardio-protective mechanism is warranted. Similarly, ACE2 receptors are also expressed in the cerebral cortex tissue, both in neurons and glia. Therefore, it seems very possible for both cardiovascular and cerebrovascular systems to be damaged leading to further dysregulation and increased risk of mortality risk. This review aims to discuss the current literature related to potential complications of COVID-19 infection with hypertension and the vasculature, including the cervical one. Finally, age is a significant prognostic indicator among COVID-19 patients. For a mean age group of 70 years, the main presenting symptoms include fever, shortness of breath, and a persistent cough. Elderly patients with cardiovascular comorbidities, particularly hypertension and diabetes, represent a significant group of critical cases with increased case fatality rates. With the current understanding of COVID-19, it is essential to explore the mechanisms by which SARS-CoV-2 operates to improve clinical outcomes for patients suffering from underlying cardiovascular diseases and reduce the risk of such conditions *de novo*.

## Introduction

On December 8, 2019, a novel coronavirus outbreak was reported in the capital of China’s Hubei province, Wuhan. Pathologically coined SARS-CoV-2 (Severe Acute Respiratory Syndrome Coronavirus 2), COVID-19 was declared a global pandemic by the World Health Organization (WHO) on March 11, 2020. Since its emergence, the epicenters shifted dramatically from China, Italy, and then to the United States. As of December 26, 2020, the virus has spread to 218 countries and territories, with the highest prevalence in the United States, India, Brazil, Russia, and France. With over 80.4 million confirmed cases, the worldwide death toll has surpassed 1.7 million. The high infectivity, low virulence, and asymptomatic transmission present an unprecedented challenge to healthcare professionals. The social, medical, and economic impact of this global health emergency represented a crisis like no other in recent decades.

In general, there are seven known coronaviruses capable of infecting humans. Alphacoronaviruses, such as HCoV-NL63 and 229E, are associated with mild disease in infected adults. Betacoronaviruses, such as Middle East Respiratory Syndrome (MERS) and Severe Acute Respiratory Syndrome (SARS), lead to severe respiratory illness, while OC43 and HKU1 tend to bring about mild symptoms. The novel coronavirus, SARS-CoV-2, is an enveloped, positive-sense, single-stranded, RNA betacoronavirus, belonging to the *Coronaviridae* family.

Betacoronaviruses contain glycosylated viral spike (S) proteins, which function in host infectivity’s entry mechanism. The S glycoprotein contains two distinct subunits required for viral entry, namely S1 and S2. The S1 domain is the receptor-binding domain, and the S2 subunit is necessary for viral fusion into the host cell. The S1 subunit of SARS-CoV-2 closely resembles a pangolin-originating virus, while the trimeric S2 domain is characteristically similar to its counterpart in the bat betacoronavirus, RatG13 (Segreto and Deigin, 2021). The SARS-CoV-2 S glycoprotein binds to the angiotensin-converting enzyme 2 (ACE-2) receptor protein with a higher affinity than SARS-CoV. This is due to a higher number of binding site interactions (Wrapp et al., 2020). There is further speculation that an RRAR furin cleavage site, unique to the S glycoprotein of SARS-CoV-2, significantly increases tissue tropism, virulence, and transmissibility (Xia et al., 2020). For viral entry into the host cell, the protein must be primed for cleavage at both S1/S2 and at the S2’ cleavage site, within the S2 subunit. This occurs sequentially with furin cleaving at S1/S2 and TMPRSS2 cleaving at S2’. With both cleavage events required for infection, inhibition of either prevents viral fusion with the host cell. Furthermore, there is an unusual synonymous to non-synonymous mutation ratio in nucleotides’ distribution after the furin cleavage site (Wang et al., 2021). This oddity may indicate a recombination event of bat or pangolin origins. Such unique structural characteristics of the S glycoprotein present obstacles for biomedical researchers. Once inside the host cell, RNA replication is carried forward by RNA-dependent RNA polymerase. This process encodes six open reading frames and involves discontinuous sub-genomic mRNA transcription.

The reproduction rate of SARS-CoV-2 at 2-2.5 is significantly higher than that of SARS-CoV. Additionally, the virus tends to remain on contaminated surfaces for a more significant duration while also producing greater loads of virions in infected hosts than SARS-CoV. In fact, SARS-CoV-2 remains up to three days on plastics and stainless steel, one day on cardboard, and up to four hours on copper following droplet application indicates the long-term resistance of this novel coronavirus (NIH, 2020). Not surprisingly, severe illness due to SARS-CoV-2 is attributed to an increased viral load of virion production and a longer duration of virus shedding. This can provide a quantifiable indication of the prognosis of critically ill patients.

The pathogenesis of SARS-CoV-2 is highly linked to a disproportionate production of cytokines as a direct response of the immune system. Cytokines normally function to regulate innate and adaptive immune processes to control infectious agents. These membrane-bound or secreted glycoproteins are found in massive concentrations in COVID-19 patients due to a cytokine release syndrome (CRS). Such exaggerated release of proinflammatory molecules causes tissue damage and may present with a broad range of symptoms caused by hyper-inflammation, from pneumonia to acute respiratory distress syndrome (ARDS). In essence, the CRS exemplifies how a protective immune response can become extremely harmful when exacerbated and amplified. Overall, the intertwining of these cytokines and chemokines causes immune cells to become hyperactive which is highly detrimental to health (Darif et al., 2021). Table 1 highlights the role of the main cytokines involved in COVID-19 pathogenesis.

**TABLE 1:** Cytokine pro-inflammatory role in COVID-19 pathogenesis (Darif et al., 2021).

Cytokine	Pro-inflammatory role in COVID-19 pathogenesis
IL-1β	*Produced by macrophages, monocytes, dendritic cells, B lymphocytes, neutrophils, and synovial fibroblasts* Promote production of IL-6 and other hematopoietic factors Induces production of cyclooxygenase 2 (COX-2), phospholipase A (PLA), and inducible nitric oxide synthase (iNOS) to produce nitric oxide (NO), prostaglandin E2 (PGE2), and platelet-activating factor (PAF), respectively Increases expression of chemokines and adhesion molecules Elevated levels indicate pyroptosis: inflammatory lytic programmed cell death
IL-6	*Glycoprotein expressed in T and B lymphocytes, monocytes, macrophages, dendritic cells, fibroblasts, and endothelial cells* Stimulate growth/differentiation of B lymphocytes Increases platelet production Activates hepatocytes to secrete C-reactive protein (CRP) and fibrinogen Regulates hematopoiesis, immune system, and inflammation
IL-8 (CXCL8)	*Produced by monocytes, macrophages, endothelial cells, epithelial cells, and airways smooth muscle cells* Chemotactic and priming effect on neutrophils, mediates inflammation Bactericidal effect by activating reactive oxygen species (ROS) production Elevated in severe COVID-19 cases Induces neutrophil extracellular traps (NETs), netosis
IL-17	*Produced in the lung by TH17 cells, as a response to viral infection* Induces chemokine production to recruit leukocytes Induces the expression of pro-coagulation factors when in combination with TNF-α Increased thrombosis increases the risk for cardiovascular-related illness Inhibits apoptosis of virally infected cells, promoting viral replication Increased TH17 cells with high CD8 + T cell toxicity, involved in ARDS
TNFα	*Produced by macrophages, mast cells, T lymphocytes, epithelial cells, airway smooth muscle cells* Causes bronchial hyperresponsiveness, neutrophil recruitment, and inflammation Induces T lymphocyte apoptosis Induces production of IL-1β and IL-6

Aside from pathophysiology, it is equally important to understand the clinical course of COVID-19. The incubation period between viral contact and the first onset of symptoms is 1–14 days (median 5.1 days). The viral shedding period is from 8–34 days (median 20 days). Within the first 11.5 days, patients mainly develop fatigue, dry cough, fever, loss of taste, loss of smell, and headache. Less common symptoms include diarrhea, loss of appetite, nasal congestion, sore throat, and rhinorrhea. The severe symptoms occur concomitantly and are often associated with bilateral pneumonia. Radiological findings typically present notable interstitial changes in mild disease and pleural effusions in severe illness. In critical patients, pneumothorax and pneumomediastinum are not uncommon (Guzik et al., 2020).

In this review, we would like to highlight that SARS-CoV-2 targets not only the respiratory tract but other vital organs as well, in particular the cardiovascular system. To better understand the association between SARS-CoV-2 and the cardiovascular system, an emphasis must be placed on pathophysiology. As previously mentioned, the viral S glycoprotein binds to the ACE-2 transmembrane receptor to facilitate viral entry and fusion. ACE-2 is highly abundant on cell surfaces of type II alveolar epithelial cells of the respiratory tract, enterocytes of the small intestine, arterial smooth muscle cells, and blood vessels’ endothelial cells. Cortical neurons and glial cells of the nervous system also express ACE-2, thereby adding to a patient’s susceptibility to neurological deficits. Infection of ACE-2-expressing pericytes can cause microvascular dysfunction, suggesting a mechanism to acute coronary syndromes. Heart failure patients tend to present with upregulated ACE-2 expression, increasing their risk for infection and severe illness. Infected patients with pre-existing hypertension are at greater risk of mortality due to vascular instability. Although these implications are prevalent for any demographic, higher mortality rates are observed in the elderly.

SARS-CoV-2 can impact the cardiovascular system in a variety of ways. Pathogenic consideration must be given to each to develop a greater understanding of a patient’s illness, associated complications, and expected prognosis. This review will go into depth on the following specific topics: (1) direct acute myocardial injury may occur as ACE-2 signaling pathways are altered upon binding of SARS-CoV-2, (2) systemic inflammation may result as characterized by cytokine storms and acute systemic inflammatory responses, (3) systemic infection and hypoxemia caused by respiratory distress can lead to an increased myocardial demand-supply ratio, predisposing patients to acute myocardial injury, (4) systemic inflammation caused by cytokine storms will increase coronary blood flow, potentially causing plaque rupture and predisposing patients to coronary thrombosis and an eventual myocardial infarction, (5) alterations in heart function leading to arrhythmias, ventricular dysfunctions, and heart failure, (6) adverse effect of antiviral therapies on the cardiovascular system, (7) electrolyte imbalances, particularly hypokalemia predisposing patients to tachyarrhythmias, and (8) the subsequent impact on geriatric care of patients with underlying CVDs (Bansal, 2020).

Patients with pre-existing cardiovascular conditions are at significantly greater risk of severe illness due to SARS-CoV-2. This review also evaluates the highest contributing factors, such as pre-existing hypertension, diabetes, cardio-cerebrovascular disease, and a history of underlying heart conditions. Essential to mention, SARS-CoV-2 may also precipitate *de novo* cardiac complications. The interplay between existing cardiac conditions and *de novo* cardiac complications is the focus of this review. As more clinical outcome data become available, our understanding of SARS-CoV-2 will evolve. With the high pace of constant research on COVID-19, the study aims to aggregate, analyze, and present the knowledge currently available.

## Hypercoagulation/Thrombosis

SARS-CoV-2 can clinically manifest with a broad range of respiratory presentations. From upper airway symptoms to fatal acute respiratory distress syndrome (ARDS). Elderly and immunocompromised patients are at significantly greater risk of more terminal, progressive hypoxemia-related illness and require mechanical ventilation. ARDS presents with a peripheral ground-glass appearance on a chest x-ray (CXR) and computed tomography (CT) imaging. The histologic, hallmark presentation of ARDS is diffuse alveolar damage with intra-alveolar fibrin deposition causing the formation of a hyaline membrane (Katzenstein et al., 1976). As discussed in detail in this section, there are characteristic vascular changes associated with SARS-CoV-2 infection. Patients present with increased d-dimer levels, more formation of fibrin thrombi, and are at greater risk of diffuse intravascular coagulation (DIC). DIC is highly associated with multisystem organ failure and a high mortality rate (Porfidia and Pola, 2020; Tang et al., 2020; Zhu et al., 2020).

In a 2020 autopsy study conducted by Ackermann et al. (2020), the morphological and molecular distinctions of SARS-CoV-2 infection were analyzed. Comparisons were made between 3 groups: patients with COVID-19, influenza A, and unaffected control group. The first distinctive feature was severe endothelial injury. This is associated with diffuse alveolar damage and increased infiltration of lymphocytes, particularly elevated CD4 + T cells. The second observation from this study was widespread thrombosis. The prevalence of alveolar microthrombi was 9 times greater in COVID-19 patients as compared to influenza. Further examination with 3D microCT showed complete occlusions of precapillary and postcapillary blood vessels. The third significant finding by Ackermann et al. was intussusceptive angiogenesis in COVID-19 patients, characterized by pillars spanning the vessel lumen. Although hypoxia was present in patients with both COVID-19 and influenza, the increased susceptibility to form microthrombi and increased endothelial damage from SARS-CoV-2 is thought to contribute to this angiogenesis (Ackermann et al., 2020). The remainder of this section will continue to highlight vascular pathogenesis, and will then focus on hypercoagulation and thrombosis.

The vascular endothelium has proved to be intimately involved in the pathogenesis of SARS-CoV-2 (Ackermann et al., 2020; Evans et al., 2020). The vascular endothelium warrants discussion in this review not only because it is the layer of cells that separate the blood from underlying tissues, but also its role in maintaining cardiovascular equilibrium. As a result, the degree of endothelial dysfunction may have implications on the severity and prognosis of patients infected with COVID-19 (Klok et al., 2020). A key mechanism that contributes to vascular endothelial dysfunction is the cytokine storm that occurs with SARS-CoV-2 infections (Chen T. et al., 2020; Chen X. et al., 2020; Huang et al., 2020). Important inflammatory cytokines involved in the storm include IL-1 and IL-6, which cause endothelial damage ultimately leading to increased vascular permeability and thrombosis (Chen X. et al., 2020; Evans et al., 2020; Huang et al., 2020; Klok et al., 2020).

Evans et al. (2020) suggests that the propensity for COVID-19 to cause thrombosis occurs due to the prothrombotic state that stems from the disparities between thromboxane vs. prostacyclin synthesis from the vascular endothelium. Furthermore, the review indicates that the prothrombotic/proaggregatory state could also be explained by the platelet-induced enlistment of leukocytes to the endothelial surface and the formation of endothelial-platelet-leukocyte aggregates. A study performed by Zheng et al. (2021) used animal model K18-hACE2 mice to study SARS-CoV-2 pathogenesis via intranasal administration of SARS-CoV-2 at varying plaque-forming units (PFU). Upon observation of various organs post-inoculation, they observed infected lung tissue had extensive alveolar damage including vascular thrombi, cell death, and immune cells (macrophages). Fibrin thrombi in the liver were also noted which is consistent with known coagulopathy effects of COVID-19, these changes were not observed in other organs (heart, spleen, kidney, small intestine, colon). These findings by Zheng et al. (2021) in conjunction with Evans et al. (2020) are consistent with the current understanding of the propensity for the SARS-CoV-2 virus to cause thrombosis, however, the exact mechanism by which this occurs has yet to be elucidated.

In addition, a key feature of endothelial cells to consider when studying COVID-19 is endothelial pericytes. Pericytes are the supportive cells of the vascular endothelium (Attwell et al., 2016) and have been shown to play a critical role in SARS-CoV-2 endothelial pathogenesis (Evans et al., 2020; He et al., 2020). Specifically, the loss of pericytes may play a central role in vascular dysfunction and hypercoagulability often seen in cases of COVID-19 (Evans et al., 2020; He et al., 2020). A paper published by He et al. (2020) indicates that the association between the SARS-CoV-2 virus and pericytes may come from pathological vascular endothelial disruption via underlying conditions that affect vascular permeability such as hypertension. The mechanism by which pericytes are lost is still unclear, but studies have suggested that apoptosis is likely a key mechanism involved (Cardot-Leccia et al., 2020).

**FIGURE 1:**
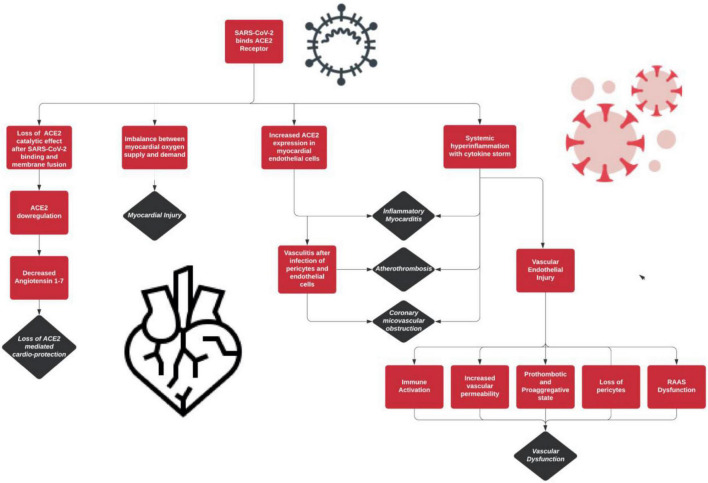
SARS-CoV-2 effects on the cardiovascular components and associated complications.

The SARS-CoV-2 infection has distinctive effects on the vasculature of the human body. The different components of the vasculature and the effects of the virus on each are highlighted in Table 2. In addition, Figure 1 provides an overview of these distinctive features of COVID-19’s pathogenesis.

**TABLE 2:** SARS-CoV-2 effects of vascular components and associated complications.

Vascular Components	COVID-19 induced effects	Complication(s)	Refs.
*Endothelial Cells*	Virus infection initiates complement activation. Induction of proinflammatory markers causing inflammation and endothelial injury. Endothelial injury causes activation of platelets and clotting factors. Pericyte disruption/dysregulation.	Hypercoagulability/Thrombosis/MI Myocardial Injury Atherosclerosis Hypertension	Lazaridis et al., 2020; Perico et al., 2021; Varga et al., 2020
*Platelets/Clotting factors*	Induction of MAPK pathway. Increased MPV indicates hyperactivity of platelet aggregation. Elevated FV and FXIII. Elevated fibrinogen, fibrin degradation products, d-dimer, vWF.	Hypercoagulability/Thrombosis	Zhang S. et al., 2020; Siddiqi et al., 2021
*Electrical Conduction*	Hypoxia-induced ARDS leads to changes in the timing of depolarizations and action potentials. Electrolyte derangement: hypokalemia, hyponatremia, hypomagnesemia, and hypocalcemia. Infection severity seems to correlate with the risk of the onset of new cardiac arrhythmia.	Arrhythmia	Madjid et al., 2019; Bhatla et al., 2020; Chen D. et al., 2020; Di Filippo et al., 2020; Lippi et al., 2020a
*Immune Dysregulation*	Cytokine storm, macrophage activating storm. IL-1, IL-6, damage-associated molecular patterns (DAMPS), pathogen-associated molecular patterns (PAMPS) activate endothelial cells. Activated endothelial cells induce proinflammatory gene expression and immune response (leukocyte recruitment, endothelial permeability). NETosis: Activated neutrophils form neutrophil extracellular traps (NETs), leading to pathogen response and clotting.	Immune Exhaustion Hyperinflammatory Syndrome Myocardial Injury	Lazaridis et al., 2020; Siddiqi et al., 2021
*RAAS Dysregulation*	ACE-2 facilitates the cleaving of angiotensin II and counter-regulates the renin-angiotensin-aldosterone system. SARS-CoV-2 decreases ACE-2 expression by ADAM17 mediated cleavage of ACE-2, causing reduced endothelial cell protection. COVID-19 patients show increased serum levels of angiotensin II, a potent vasoconstrictor that induces inflammation, fibrosis, and hypertrophy. Angiotensin II regulates NADPH oxidase activity, leading to increased production of reactive oxygen species (ROS), which further damages the endothelium. Downregulation of Angiotensin 1-7 (cardioprotective factors). Elevated Angiotensin II-induced hypokalemia and elevated blood pressure.	Myocardial Injury Hypertension Oxidative Stress	Nguyen Dinh Cat et al., 2013; Chen T. et al., 2020; Lazaridis et al., 2020

Critically ill COVID-19 patients often present with hypercoagulation conditions as a significant complication. Recent research suggests an association between SARS-CoV-2 infection and secondary complications such as thrombocytopenia, thrombosis, sepsis, and disseminated intravascular coagulation. Although the underlying mechanisms remain elusive, several factors may contribute to such a link. Platelets may be activated by the overproduction of cytokines, which may induce a pro-inflammatory state—thereby triggering the complement cascade. Endothelial activation may play a role in platelet activation. After lysis caused by SARS-CoV-2, endothelial cells may release inflammatory factors that may induce the complement cascade. An unbalance of angiotensin II-angiotensin II receptor signaling could also be a possible mechanism to hyper-platelet activation, as could hypoxemia be caused by lung injury. Wide-scale clinical studies indicate that between 19 to 36% of SARS-CoV-2 patients present with thrombocytopenia on initial assessment. Further analysis reveals that 31% of SARS-CoV-2 patients admitted to the ICU presented with thrombosis-related complications compared to a mere 1.3% of the non-ICU group. It is reasonable to assume a correlation between severe illness caused by SARS-CoV-2 and the incidence of thrombotic complications. This section of the review aims to understand the mechanisms that may lead to such a hypercoagulable state (de Vries, 2020).

Platelets, also termed thrombocytes, are the blood component responsible for blood clotting and clumping at the site of blood vessel damage. Derived as fragments of megakaryocytes from the bone marrow, platelets undergo a dynamic activation process under the complement cascade. This complex process involving over 193 proteins and 300 interactions between factor proteins is called hemostasis. It has three distinct steps to form the platelet plug: adhesion, activation, and aggregation. Platelet disorders are classified into three broad categories: (1) lower than normal platelets, termed thrombocytopenia, (2) dysfunctional platelets, which may be congenital or acquired, and (3) higher than normal platelets, termed thrombocytosis/thrombocythemia.

Regardless of the underlying mechanism, platelets are the critical component involved in thrombotic complications of SARS-CoV-2. Upon infection and activation, they aggregate on the sub-endothelium forming a thrombus. As noted in SARS-CoV-2 patients, hyperactive thrombosis increases the risk for arterial ischemia, pulmonary embolisms, and myocardial infarctions. Much of the current understanding of this SARS-CoV-2 is based on outbreak-causing viruses of the past. HIV, HCV, influenza, Ebola, and DV all directly caused the hyperactivity of platelets. Their mechanisms to activate the coagulation cascade presents a starting point for deciphering the direct effects of SARS-CoV-2 on hypercoagulation.

On the note of evolving research, it is essential to mention that data collected from the SARS and MERS epidemics holds high value in understanding SARS-CoV-2. The effect of this novel coronavirus on the complement system is unique yet similar in many respects. As our knowledge evolves, it remains essential to draw such correlations to develop treatments. The complement system is an integral and innate component of the immune system. The classical, lectin, and alternative are three pathways by which the complement system is activated. During this cascade of cleavages, pro-inflammatory precursors stimulate phagocytes, induce further inflammation, and trigger a cell-killing complex. This innate immune response is responsible for clearing pathogens and infections; however, the detriment on bodily tissues can cause severe harm when left unchecked. Previous studies show SARS-CoV as capable of binding mannose-binding lectin (MBL) to initiate the lectin complement pathway. The alveolar epithelial and pneumocytes of SARS-CoV-2 patients present markedly increased expression of MBL, C3, C4, and C5b-9 complement proteins. Elevated levels of plasma complement activation proteins were particularly noted in patients with severe COVID-19 illness (Perico et al., 2021).

The activation of complement pathways triggers endothelial layer injury and subsequent cell death. The clotting cascade is activated following vascular denudation exposes the underlying basement membrane. COVID-19 patients with severe illness present with microvascular thrombosis, internal edema, inflammation, and hemorrhagic sequelae caused by previous events. SARS-CoV-2 autopsy studies showed small vessel thrombosis associated with alveolar hemorrhage. As further investigations continued to show small vessels with platelet-fibrin thrombi, it became clear that thrombosis and coagulopathy are possible outcomes of SARS-CoV-2 infection (Perico et al., 2021). Considering acute pulmonary embolisms are life-threatening complications in COVID-19 patients, it is imperative to learn more about the causative mechanisms.

In a thorough study conducted by Zhang S. et al. (2020), it was noted on admission that critically ill patients presented with abnormal platelet parameters. Patients presented with reduced platelet counts and plateletcrit (PCT) and increased mean platelet volume (MPV) and platelet distribution width (PDW). Abnormal findings also included elevated d-dimer, fibrinogen degradation products (FDP), increased prothrombin time (PT), activated partial thromboplastin time (APTT), international normalized ratio (INR), and decreased prothrombin time activity (PTA) compared to healthy donors, non-COVID-19 patients, or COVID-19 patients with mild symptoms only. Admitted patients that did present with thrombocytopenia also presented with elevated PT, INR, APTT, d-dimer, and FDP. Increased MPV is an indicator of platelet hyperactivity. Integrin αIIbβ3 activation, or PAC-1 binding, and platelet expression of P-selectin were increased as well, most significantly noticeable in critical patients. These results indicate hyperactivation of platelets, paired with platelet consumption, hence the thrombocytopenia. Higher levels of PAC-1 binding and P-selectin expression were also paired with the presence of viral RNA in blood samples. Zhang S. et al. (2020) tested the effect of SARS-CoV-2 RNA-positive blood on platelet aggregation. As hypothesized, increased platelet aggregation was noted in blood samples infected with SARS-CoV-2 RNA. This association indicates SARS-CoV-2 RNA as an indicator of hyperactivity of platelet aggregation.

For SARS-CoV-2 to infect platelets, platelets must express both ACE-2 and TMPRSS2. RT-PCR and Western blot analysis by Zhang S. et al. (2020) confirmed that ACE-2 was just as prevalent on the surface of platelets as compared to human colon cell line Caco-2 and lung cell line Calu-3. TMPRSS2 expression was further confirmed by flow cytometry and confocal immunofluorescence. The next question was whether SARS-CoV-2 directly induces the activation of platelets. Although incubating SARS-CoV-2 with washed human platelets did not induce platelet aggregation, a dose-dependent response was observed when thrombin, collagen, and ADP were added to the mixture. While SARS-CoV-2 may not directly increase platelet aggregation, the virus could infect platelets via the ACE-2 entry receptor. As the virus enters the platelet, ACE-2 is internalized and degraded, causing a decrease in surface ACE-2 receptors on platelets. This was consistent with the researchers’ findings, particularly for critical patients. With the known association between SARS-CoV-2 and ACE-2 receptors on platelets, the next step of learning about the underlying mechanisms is to learn more about the S-glycoprotein interaction. Zhang S. et al. (2020) further found that the S1 subunit alone was responsible for increasing platelet aggregation, enhanced αIIbβ3 activation, and P-selectin expression. This indicates that the binding of the S1 is responsible for platelet regulation.

Previous studies report that the mitogen-activated protein kinase (MAPK) is stimulated in platelet activation and thrombosis. Further studies indicate that activation of the ACE-2 and MAPK pathway has a moderating effect on the cytokine-induced systemic inflammation from SARS-CoV-2. Zhang S. et al. (2020) deduced that the MAPK pathway is activated after the ACE-2 binding. Increased phosphorylation of MAPK, Erk, p38, and JNK in platelets of SARS-CoV-2 patients was noted. These results show that SARS-CoV-2 directly activates the ACE-2 and MAPK pathway, increasing platelet activation. Considering platelet activation is potentiated with the activation of the ACE-2/MAPK pathway, Zhang S. et al. (2020) sought to determine if this enhanced the thrombosis potential. On conducting a mice study, it was found that the SARS-CoV-2 S glycoprotein did potentiate thrombosis in the mice group with platelets.

As earlier mentioned, a coagulation cascade is responsible for the activation and aggregation of platelets. Zhang S. et al. (2020) furthered their investigation by determining the effect of SARS-CoV-2 on the secretion of coagulation factors, cytokines, and platelet-leukocyte aggregates (PLA). The study found that SARS-CoV-2 directly amplified the secretion of FV and FXIII. The levels observed were similar to those present during thrombosis. The α-subunit of platelets is capable of releasing several inflammatory cytokines. These mediators are secreted upon activation and are key players of the subsequent coagulation steps and immune response. The S-glycoprotein was found to increase plasma levels of inflammatory cytokines PF4, TNF-α, IL-8, and IL-1β. Notably, the plasma levels were highest in patients with severe SARS-CoV-2 illness. Furthermore, changes in PLA formation due to SARS-CoV-2 were assessed infection as PLA formation is often increased in inflammatory and thrombotic conditions and can be attributed to amplified neutrophil and platelet activation. P-selectin is a cell adhesion molecule (CAM) protein mediating the interplay between platelets and neutrophils. P-selectin expression was markedly increased following SARS-CoV-2 treatment, thereby increasing the formation of PLAs. Therefore, a clear association became present between infection from SARS-CoV2 and a marked rise in plasma coagulation factors, cytokines, and PLAs, increasing a patient’s predisposition to inflammatory and thrombotic conditions.

As a final stage of the study, the effect of a human recombinant ACE-2 protein and S-glycoprotein antibody treatment on levels of platelet spreading and clot retraction was evaluated. As expected, both the ACE-2 protein and S-glycoprotein antibody reduced infection of SARS-CoV-2. Platelet spreading and clot retraction were notably reduced, as were levels of S-glycoprotein-induced thrombosis at an injury site. This experiment was conclusive to directly associate platelet activation and the activation of ACE-2 receptors with the S-glycoprotein of SARS-CoV-2. These studies shed light on numerous interactions between the virus and factors involved in thrombosis and inflammation via the S-glycoprotein/ACE-2 interaction. As research evolves, antiplatelet therapies may be considered for patients at higher risk of hypercoagulation secondary to COVID-19 (Zhang S. et al., 2020).

## Cardiac Arrhythmias

Cardiac arrhythmias are broadly defined as heart rhythm defects when the electrical impulses that synchronize the heartbeats are abnormal, causing the heart to beat too fast, slow, or irregularly. Arrhythmias have severe implications on the heart’s effectiveness to pump blood effectively throughout the body and are mainly an issue as they can have adverse effects on other organs. Furthermore, this is of grave concern in the current climate of individuals infected with SARS-CoV-2, both with and without underlying cardiovascular issues. The mechanisms by which arrhythmogenicity in viral infections, in general, are through the interplay between host factors and viral characteristics. The knowledgebase of arrhythmia complications resulting from COVID-19 is in its early stages. Still, the novel coronavirus’s current understanding to cause cardiac arrhythmias is rapidly evolving, with various forms of arrhythmias documented (Dherange et al., 2020). This review will concentrate on viral epidemics of the past, on the causes of COVID-19 induced cardiac arrhythmias, the various forms of arrhythmias that result, and disparities between Non-ICU vs. ICU patients.

The bulk of the current knowledge base regarding COVID-19 largely stems from past epidemics, including; SARS-CoV, MERS-CoV, and Influenza. SARS-CoV-2, similar to previous viral epidemics, has shown significant associations with cardiovascular disease, poor prognosis, and outcomes. During the SARS-CoV epidemic, there were many sinus tachycardia cases, sinus bradycardia, and palpitation in the form of tachycardia noted. Yu et al. (2006) showed that amongst a cohort of 121 admitted patients, sinus tachycardia was by far the most common cardiovascular finding associated with SARS-CoV infection at a rate of 72%. The tachycardia seen in the patient cohort continued for roughly 12.7 days with a mean heart rate of 117 beats/min and continued in 40% of patients within 30 days after discharge from the hospital. Furthermore, sinus bradycardia was noted in 18 (14.9%) of the cohort’s 121 patients. However, unlike sinus tachycardia, sinus bradycardia was transient with a mean duration of 2.6 days and a mean heart rate of 43 beats/min. Lau et al. (2005) reported heart palpitations in the form of tachycardia at rest or with mild exertion, which they indicated was possibly secondary to impaired cardiac function and arrhythmias.

The MERS-CoV epidemic of 2012 was much more dangerous than the SARS epidemics, with a mortality rate of 60% with complications including cardiac arrhythmias, specifically tachyarrhythmias and severe bradyarrhythmias eventually requiring pacemakers was noted in 15.7% of patients (in a case series of 70) (Saad et al., 2014). It was well documented that these complications among the cohort of 70 were more likely to occur amongst individuals with underlying cardiovascular disease (Saad et al., 2014).

Lastly, influenza cannot be dismissed in this discussion as it has been well known to cause cardiac arrhythmias. Studies have shown that influenza can cause various tachyarrhythmias, ventricular fibrillation, and heart block (Atri et al., 2020). Also, Madjid et al. (2019) noted that patients with implantable cardioverter-defibrillator or resynchronization therapy with a defibrillator had more shocks administered during influenza season than other periods throughout the year. Although influenza is not amongst the coronavirus family like the SARS and MERS virus, its association with arrhythmias is a worthwhile model to extrapolate and help comprehend the novel coronavirus, SARS-CoV-2. Much more investigation is needed in understanding the association between COVID-19, cardiovascular disease, and cardiac arrhythmias. However, arrhythmias that manifested in previous viral epidemics can be a useful source by which the current understanding of the link between the novel SARS-CoV-2 virus and cardiac arrhythmias in patients with pre-existing cardiovascular conditions and poor outcomes in patients can be built upon. Regardless, viral infection in patients with chronic cardiovascular diseases may have an increased probability of becoming unstable and have deleterious myocardial effects and should be closely monitored.

There have been numerous potential mechanisms discussed in the literature regarding the development of cardiac arrhythmias stemming from SARS-CoV-2 infection. Cardiac arrhythmias in the setting of COVID-19 seem to be more prevalent in patient populations with underlying cardiovascular conditions due to myocardial damage and increased inflammatory response (Madjid et al., 2019). Studies have shown that arrhythmias may occur due to a variety of different mechanisms (Lazzerini et al., 2019; Madjid et al., 2019; Bhatla et al., 2020). However, this review will focus on hypoxia caused by viral tissue involvement of lungs, abnormal host immune response, and electrolyte derangement.

It is known that COVID-19 causes hypoxia as the virus can lead to new-onset acute respiratory failure that occurs due to viral-induced lung injury (Madjid et al., 2019). The decreased oxygen conditions in the body will lead to anaerobic glycolysis in cells, subsequently reducing the intracellular pH and increasing cytosolic calcium levels. This will not only lead to changes in the timing of action potentials but will also lead to changes in the timing of depolarizations (early/late) (Madjid et al., 2019). Furthermore, hypoxia can also lead to an increased concentration of extracellular potassium. This ultimately decreases the depolarization threshold, accelerating biochemical and electrical conduction which disrupts the heart’s synchrony leading to new-onset cardiac arrhythmias in patients.

SARS-CoV-2 can lead to arrhythmias also through its ability to elicit an exaggerated host immune response via cytokine-storm-related cell death. In particular, cytokine-storm can lead to myocardial damage which may play a crucial role in developing cardiac arrhythmias (Driggin et al., 2020). The cytokine players that have been demonstrated to act in this regard include IL-6, tumor necrosis factor α, and IL-1. These cytokines and particularly IL-6, have been shown to extend the ventricular action potential by modifying cardiomyocyte ion channels and ultimately lead to QTc interval prolongation (Lazzerini et al., 2015; Lazzerini et al., 2020). Thus, they recommend anti-IL-6 therapies to patients with SARS-CoV-2 infection to prevent myocardial damage and reduce the risk of cardiac arrhythmia complications.

Finally, the electrolyte derangements that cause arrhythmias have been well investigated (Surawicz, 1966). In particular, studies have shown that COVID-19 infection can lead to various electrolyte changes, including hypokalemia, hyponatremia, hypomagnesemia, and hypocalcemia (Chen D. et al., 2020; Lippi et al., 2020a). In a study performed by Chen D. et al. (2020), they looked at a patient population of 175 with SARS-CoV-2 infection. They found that 62% of the patients had potassium < 3.5 mmol/l, and of the 62, 22% had severe hypokalemia (potassium < 3.0 mmol/l). It should be noted that this study found that there was a positive correlation between hypokalemia and the severity of SARS-CoV-2 infection. Potassium’s significance cannot be overlooked as it is known to play an essential role in preventing myocardial injury and appropriate potassium levels are necessary to avoid potential cardiac arrhythmias. It has been reported that hypokalemia can cause several different types of arrhythmias, including ventricular fibrillation, polymorphic VT, and Torsades De Pointes (TDP) as a result of changes to the heart conduction velocity (Skogestad and Aronsen, 2018).

COVID-19 infection has also been noted to cause hypocalcemia (Di Filippo et al., 2020; Lippi et al., 2020a). Di Filippo et al. (2020) performed a retrospective study of patients in the Emergency Department in Milan, Italy. Their analysis of 531 patients found low levels of calcium in 462 (82%; Actual calcium measurement) and 414 (78.6%; Standardized calcium measurement). When comparing calcium levels between admitted vs. non-admitted patients with SARS-CoV2 infection, they discovered that individuals that were hospitalized had a lower measured calcium as opposed to the non-hospitalized counterparts (1.1 mmol/L (hospitalized) vs. 1.14 mmol/L (non-hospitalized). Hypocalcemia correlated with hospitalization and poor outcomes, including death and risk of admission to the ICU in univariate analyses of the two outcomes. The issue of the relationship between COVID-19 infection and hypocalcemia can be a concern for adverse cardiac consequences, including ventricular arrhythmias (Ashwin Reddy, 2019) and cardiac arrest (Yarmohammadi et al., 2017).

A study done by Bhatla et al. (2020) monitoring a cohort of 700 patients with COVID-19 over 74 days showed significant differences in the onset of cardiac arrhythmias between ICU and Non-ICU patients. The study indicated that compared with non-ICU patients, patients admitted to the ICU were older, were more likely to suffer from underlying cardiovascular conditions, and had lower oxygen saturation on admission. Over the 74 days, there were a total of 53 arrhythmic events reported, with ICU, admitted patients having a > 10-fold chance of having an arrhythmic event. Furthermore, they found that the incidence of cardiac arrests in their cohort of patients correlated with the severity of the infection and had a much greater risk of developing arrhythmias. 621 (89%) of the 700 patients in the cohort admitted to the non-ICU setting had no cardiac arrest cases compared to 9 cardiac arrests amongst ICU admitted patients, further supporting the relationship between ICU vs. non-ICU admitted patients and the potential incidence of cardiac arrhythmia events.

The disparities between ICU and non-ICU patients with SARS-CoV-2 infection were also noted in a study done by Zeng et al. (2020). In a patient population of 416, they had 35 patients admitted to the ICU. Compared to non-ICU admitted patients, the ICU patients were older and have underlying cardiovascular conditions, including hypertension (37% in ICU vs. 12% non-ICU) and arrhythmias (6% in ICU vs. 0.5% non-ICU). The ICU population had more cardiac complications, including cardiac injury, atrial and ventricular arrhythmias. This may indicate that the higher incidence of cardiovascular events may stem from systemic inflammatory response or immune disorders, further intensifying the cardiac-related complications.

## Myocardial Ischemia/Injury

Myocardial ischemia/injury (MI) is a medical condition that occurs when part of the heart does not receive enough blood supply causing irreparable damage to the heart muscle, tissue, and cells. The most common causes of MI include coronary artery disease or atherosclerosis that can result in blood clots blocking blood flow of the coronary artery supplying blood to the heart. There are many different risk factors for MI including high blood pressure, diabetes, smoking, age, family history, and obesity. Amid the COVID-19 pandemic, MI has been reported in > 50% of patients who have lost their lives as a result of COVID-19 infection (Lazaridis et al., 2020). This staggering number is why this portion of the review on MI will seek to understand the intricate relationship between SARS-CoV-2 and MI. This portion of the review will look at the high ACE2 expression in myocardial and endothelial cells, systemic hyper-inflammation, the imbalance between myocardial oxygen supply and demand, loss of ACE2-mediated cardioprotection, as well as the incidence of admission for acute MI (pre-pandemic vs pandemic).

Besides dealing with economic, social, and psychosocial stress, the COVID-19 pandemic has also been shown to increase the incidence of stress cardiomyopathy. In fact, in a recent study of 1,914 patients, the effect of COVID-19 related stress was measured by looking at the frequency of stress cardiomyopathy during the pandemic and those that occurred during three previous periods. It was found that the COVID-19 caused a noticeable increase in stress cardiomyopathy, with instances rising to over four times more than usual during the months of March and April 2020. On the bright side, people with stress cardiomyopathy recover heart functions in one to two weeks, with the prognosis generally being good. Nevertheless, recurrence is at increased risk of cardiomyopathies in affected patients (Jabri et al., 2020).

It has been well established that there is high ACE2 expression seen in cardiac tissue. For this reason, SARS-CoV-2 is easily able to infiltrate cardiac cells and ultimately leads to extensive myocardial injury. ACE2 receptors are seen in cardiomyocytes, pericytes, and endothelial cells. Cardiac pericytes are particularly important in SARS-CoV-2 infection and cardiovascular pathophysiology because of their role in supporting capillary endothelial cell function. Pericyte infection by COVID-19 can disrupt the endothelial cells of the capillaries causing coronary microvascular defects and leading to extensive cardiac injury (Lazaridis et al., 2020). Varga et al. (2020) examined pathology reports of COVID-19 infections which showed evidence of diffuse endothelial infection and inflammation. These findings suggest that endothelial defects could possibly explain the destabilization of coronary plaques, clot formation, and vascular disease (Varga et al., 2020).

One of the main causes of infectious myocarditis is a viral infection. The main culprit in this is the activation of the host antiviral immune system (macrophage, natural killer cells, and T lymphocytes). A systemic hyper-inflammation is seen in patients with COVID-19 infections, a response that is characterized by increased cytokine and chemokine production (tumor necrosis factor, IL-2, IL-6, IL-7, and CCL2, etc.) (Lazaridis et al., 2020). Therefore, it is no surprise that this systemic hyperinflammatory response leads to extensive myocardial damage/injury. These findings of systemic hyper-inflammation have been further corroborated in autopsy reports noted in studies performed by Xu et al. (2020). In their investigation, they found extensive mononuclear cell infiltration in cardiac tissues of patients discovered to have severe myocarditis as well as a high viral load of the COVID-19 virus. There are few consequences of the systemic hyper-inflammation caused by the SARS-CoV-2 virus that have been noted to play a key role in myocardial ischemia/injury. This response by the body will lead to the recruitment of macrophage and leukocyte adhesion molecule expression on endothelial cells of pre-existing atherosclerotic lesions which in turn can lead to the progression of the acute coronary syndrome. It is also worth noting that the elevated levels of cytokines can lead to disruption of the endothelial cells as well as the dysfunction of the microvasculature leading to MI (Lazaridis et al., 2020).

Another possible connection that has been coined in current research regarding SARS-CoV-2 and MI is the virus’s ability to cause an imbalance of myocardial oxygen supply and demand (Lazaridis et al., 2020). Patients with COVID-19 infections often present with severe respiratory complications, hypoxia, and hypotension. Hypoxia, in particular, may play a role in the development of tissue inflammation and ultimately cause myocardial damage (Eltzschig and Carmeliet, 2011). In addition, hypotension can also lead to a reduction in oxygen supply to the heart. COVID-19 has been noted to lead to hypotension as a result of its ability to cause fever, sepsis, and cytokine storm (systemic hyper-inflammation) (Libby, 2020). A consequence of systemic inflammation/infection increases the metabolic demand of the myocardial cells as well as other peripheral tissues in the body (Musher et al., 2019). These relationships suggest that there may be a relationship between COVID-19 and MI due to its pathophysiological effects in creating an imbalance of myocardial oxygen and demand.

Lastly, ACE2 is a major enzyme in the renin-angiotensin system (RAA system) that catalyzes the conversion of angiotensin II to angiotensin 1-7. Angiotensin 1-7 takes on cardioprotective effects including antiarrhythmic, anti-remodeling (Patel et al., 2016), antiproliferative on cardiac fibroblasts (McCollum et al., 2012). The internalization of ACE2 via SARS-CoV-2 binding leads to the loss of these protective and external ACE2 catalytic effects (Lazaridis et al., 2020). Thus, patients with COVID-19 infections will experience a decreased availability/downregulation of ACE2. This in turn favors the development of atherosclerosis due to the buildup of angiotensin II as well as the loss of cardioprotection via insufficient angiotensin 1-7 ultimately resulting in myocardial compromise (Lazaridis et al., 2020). The involvement of ACE2 in causing MI can be further supported as research shows that SARS-CoV-2 disrupts the RAA system by elevating angiotensin II which leads to hypokalemia and elevated blood pressure (Chen T. et al., 2020).

**FIGURE 2:**
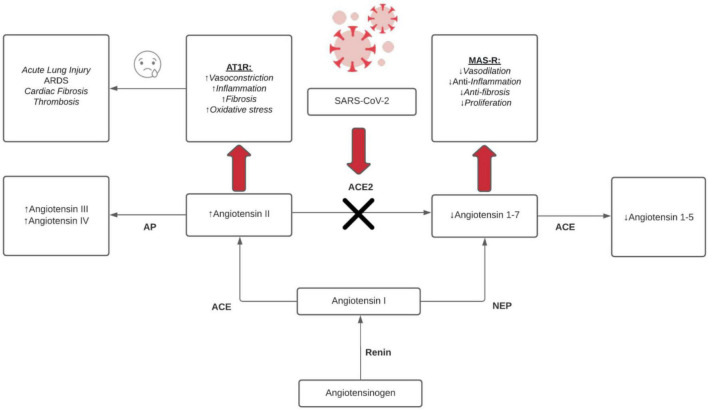
RAAS dysfunction effects from COVID-19. ↑ = upregulation/increase and ↓ = downregulation/decrease, red arrow = binding, black X = inhibition, black arrows = conversion/metabolism.

Figure 2 outlines the intricate relationship between SARS-CoV-2 and the ACE2 receptor. The renin-angiotensin-aldosterone system (RAAS) pathway initially begins with the conversion of angiotensinogen to angiotensin I via renin. Angiotensin I can then form angiotensin II and angiotensin 1-7 via the angiotensin-converting enzyme (ACE) and endopeptidases (NEP), respectively. Angiotensin II is either (1) metabolized to angiotensin III/IV by aminopeptidases (AP) (2) binds ANGII type 1 receptor (AT1R) (3) or converted to angiotensin 1-7 by ACE2. On the other hand, angiotensin 1-7 is (1) metabolized to angiotensin 1-5 (2) or binds Mas receptor (MAS-R). Emphasis is placed particularly on the ACE2-SARS-CoV-2 interaction. The binding of COVID-19 to ACE2 enzymes causes a shift of this pathway particularly in upregulating angiotensin II and associated interactions. As noted in Figure 2, angiotensin II binding AT1R promotes vasoconstriction, inflammation, fibrosis, oxidative stress. These processes lead to known complications of COVID-19 including acute lung injury, ARDS, thrombosis, cardiac fibrosis. This system, under normal conditions, is safeguarded by the Angiotensin 1-7 binding of MAS-R that promotes protective factors including vasodilation, anti-inflammation, anti-fibrosis, and decreased proliferation. Overall, this figure shows a clear imbalance of the RAAS system by COVID-19 and shows that key cardioprotective factors are lost in SARS-CoV-2 infections (McCollum et al., 2012; Patel et al., 2016; Chen T. et al., 2020; Lazaridis et al., 2020; South et al., 2020).

Given what is known about SARS-CoV-2 and its harmful effects on the heart and consequently its proclivity to cause MI, there has been a surprising trend reported in the literature of a decline in hospital admissions for acute MI pre-COVID-19 vs. pandemic (Solomon et al., 2020). Solomon et al. (2020) performed a study on Kaiser Permanente Northern California data of 21 medical centers and 255 clinics. In the study, they looked at the incidence of hospital admission for acute MI (NSTEMI and STEMI) pre-pandemic vs. pandemic time periods. They found an overall decrease in admission for acute MI during the COVID-19 pandemic (January 1, 2020 - April 15, 2020). This is a curious trend that does not seem to fit current knowledge of SARS-CoV-2 and its relationship with the cardiovascular system. While there may be many reasons to explain this trend, the present understanding of COVID-19 is still in its early stages and does not offer a clear explanation at this time.

## Hypertension and Neurological Comorbidities

Hypertension or high blood pressure is a condition that presents as a long-term force of blood against the walls of the arteries and may lead to various negative health conditions later in life. Blood pressure is determined by the amount of resistance to blood flow evident in the arteries and the amount of blood pumped by the heart. High blood pressure is represented by a systolic pressure of 140 mm Hg or higher and a diastolic of 90 mm Hg or higher. Hypertension is a well-established health concern and has been associated with numerous complications including heart attack, stroke, heart failure, and aneurysms. With the recent outbreak of SARS-CoV-2, hypertension and other cardiovascular-related issues in patients have become sources of concern (Zhang and Moran, 2017). There have been numerous studies that have shown that there has been a significant association amongst patients infected with COVID-19 with hypertension and negative clinical outcomes. This portion of the review on hypertension will focus on hypertension in the setting of COVID-19 infection and RAAS inhibitors.

Hypertension is highly prevalent comorbidity in patients suffering from COVID-19. The percentage of SARS-CoV-2 patients with hypertension seen across studies ranged from 4.5% to > 30% (Tadic et al., 2020a,b). Tadic et al. (2020a), showed in a meta-analysis that COVID-19 patients had high rates of hypertension and cardiovascular disease. This has been further confirmed by studies that have shown that hypertension is one of the most common comorbidities in patients with COVID-19 infection (Zhou et al., 2020). The prevalence of hypertension among patients and the vast amount of research indicating an intrinsic relationship between hypertension and SARS-CoV-2 makes hypertension an important area of study and a topic of focus for this review.

There have been a few different roles suggested that hypertension may play in COVID-19 pathogenesis including its role as a predictor of severity of disease or leading to the worsening clinical course via acute respiratory disease syndrome or multiple organ failure. A meta-analysis of 13 studies including 2,893 patients was performed by Lippi et al. (2020b) who reported a 2.5-fold-increased risk of severe COVID-19 and increased mortality risk in patients who were infected by COVID-19 and suffer from hypertension. They also found adequate evidence showing that hypertension (both pulmonary and systemic) is associated with negative disease progression for patients with SARS-CoV-2 infection which could indicate there is a significant interplay between hypertension and COVID-19 and may ultimately leads to poor prognosis in hypertension versus normotensive patients. Although Lippi et al. (2020b) could not establish the pathophysiology between SARS-CoV-2 and hypertension, it was evident in the meta-analysis that the role hypertension plays in COVID-19 infections cannot be overlooked.

There have also been concerns regarding whether or not patients with hypertension gave them a predisposition for SARS-CoV-2 infection (Drager et al., 2020). Drager et al. (2020) notes that there is no clear evidence that patients with hypertension increase susceptibility to infection nor does it predispose them to worse outcomes. The same study also raises another concern regarding increased ACE2 expression in patients with hypertension as this could help explain worse clinical outcomes seen in patients. A study done by Pinto et al. (2020) showed elevated ACE2 expression in the lungs of patients with hypertension and presented with COVID-19 infection when compared to a control participant pool, however, no cause and effect could be established due to the cross-sectional design of the study.

Interestingly, ACE-2 receptors are robustly expressed in endothelial cells of cerebral capillaries (Pena Silva et al., 2012; Baig et al., 2020), which makes them a potential target for SARS-CoV-2. Furthermore, cortical neurons and glial cells of the nervous system also express ACE-2 that can facilitate entry of the SARS-CoV-2 (Xu and Lazartigues, 2020). The binding of SARS-CoV-2 to ACE-2 receptors in the cerebral vasculature can cause an increase in pressure leading to cerebral hemorrhage. In addition, this binding to the ACE-2 receptors can lead to a decrease in ACE-2 levels and an increase in ACE-1 levels which can lead to inflammation and injury of neurons (Roy et al., 2021). In a recent clinical report, 214 patients with SARS-CoV-2, neurologic symptoms were seen in 36.4% of patients with increase prevalence noted in patients with severe infection, with reports of acute cerebrovascular accidents and other derangements of cerebrovascular architecture (Mao et al., 2020). It has been recognized that SARS-CoV-2 has the ability to reach the cerebral vasculature through systemic circulation inducing a hypercoagulable state and hyperactive inflammatory response which can result in thrombotic events within the neuro-vasculature (Pillai et al., 2021). Particularly it is has been shown that circulating leukocytes and other factors such as angiotensin II can downregulate endothelial tight junctions (Setiadi et al., 2018). With these changes, autonomic neuropathy, characterized by abnormalities in heart rate and blood pressure, and cardiac output, is a very distinct possibility. Although the specific mechanisms leading to these downstream changes are unclear, it has been demonstrated that autonomic dysfunction can be detected through plasma catecholamine levels before cardiological abnormalities are established in suspecting persons. Catecholamine excess is also known to occur in mild to severe disease states, such as through infection with SARS-CoV-2, and leads to the progression of hypertension and subsequent end-organ damage.

It has been reported by numerous studies that SARS-CoV-2 patients with more adverse outcomes had higher rates of hypertension and cardiovascular disease. In particular, in a study of 1,590 patients done by Guan et al. (2020b), they found that diabetes, coronary artery disease, cerebrovascular disease, COPD, and cancer were most prevalent among their study population. These same diseases and complications were noted at higher rates in patients with myocardial injury with studies finding that 27.8% of patients with COVID-19 had a cardiac injury (Shi et al., 2020). Furthermore, hypertension, cardiovascular disease, and diabetes, coronary heart disease were reported to be most prevalent among lethal cases of SARS-CoV-2 (Chen T. et al., 2020; Zhou et al., 2020). Zhou et al. (2020) initially found that hypertension, diabetes, and coronary heart disease were predictors of mortality of patients with SARS-CoV-2 infection; however, this did not hold true for any of the conditions after adjusting for age.

Drager et al. (2020) investigated aging as a possible factor for worse clinical outcomes for patients with hypertension and SARS-CoV-2 infection. It is well known that elderly patients are at higher risk for COVID-19 related complications and that aging is a risk factor for developing hypertension (Zhang and Moran, 2017; Whelton et al., 2018). Guan et al. (2020a) performed an analysis across 575 hospitals investigating aging and hypertension as important comorbidities in the setting of SARS-CoV-2. They found that 16.9% of their patient population in China reported suffering from hypertension which was lower than expected. The mean age of the study population was 48 years old which for that age group in China had a prevalence of hypertension at 29.6%. They indicated that this disparity could be that patients are unaware that they had hypertension. Thus, Drager et al. (2020) concluded that based on the available literature it is clear that more research is needed in regards to the relationship between hypertension and poor COVID-19 prognosis. It cannot be concluded with certainty that patients with a history of hypertension and COVID-19 infection predisposes them to poor prognosis.

The relationship between COVD-19 and ACE2 has been a primary mechanism for investigation, particularly regarding hypertension and the effectiveness of RAAS inhibitors. There was an ongoing debate early in the outbreak as to the effect of angiotensin-converting enzyme inhibitors (ACEI) and angiotensin II receptor blockers in COVID-19 patients. Some suggested that the use of renin-angiotensin-aldosterone system (RAAS) inhibitors may exacerbate the virulence of SARS-CoV-2 (Esler and Esler, 2020; Watkins, 2020). On the other hand, there were also studies that did not find any significant association between RAAS inhibitors and SARS-CoV-2 complications (Li et al., 2020; Mancia et al., 2020; Reynolds et al., 2020). To even further complicate the debate, Meng et al. (2020) performed a study in which they reported that RAAS inhibitors may improve COVID-19 outcomes in patients with underlying hypertension.

It is now evident through a review performed by Zhang J. et al. (2020) in which they concluded that current evidence does not support concerns that the use of RAAS inhibitors leads to increased risk of COVID-19 infection or poor clinical course. Therefore, there is no indication to consider changing medications or discontinuing RAAS inhibitors at this time as this will lead to fluctuations in blood pressure and potentially exacerbate underlying diseases. However, it is still unclear if RAAS inhibitors improve the prognosis of those patients infected with SARS-CoV-2 and require further investigation.

## Geriatric Care

Senescence is associated with an eventual slowing and degradation of bodily functions. Linked to many health conditions in the elderly, reduced cardiovascular functionality, increased oxidative stress, and weakened immune defense mechanisms are not the only body processes affected. Concerning cardiology, elderly COVID-19 patients are more likely to present with reduced thrombolysis, increased risk of myocardial infarction (MI), and thereby a greater risk of heart failure. In relation to pathology, the elderly are at increased risk of contracting infectious diseases due to weakened immunity. Both age and pre-existing cardiac comorbidities are predisposing factors worth considering in patients presenting with a positive COVID-19 diagnosis (Napoli et al., 2020).

As previously discussed in this literature review, COVID-19 induces damage to the cardiovascular system in several ways. Understanding the pathogenesis and disease progression, particularly in relation to cardiovascular functions, is the first step to deciphering an ideal clinical management plan. COVID-19 patients may present with cardiac arrhythmias, myocarditis, MI, vasculitis, and pericarditis. Not only are such patients at risk of heart failure but are also predisposed to viral respiratory illness. This is due to the interdependent nature of the cardiovascular, respiratory, and immune systems. Elderly patients with a weakened immune response are at a greater risk of contracting COVID-19. This is often noticeable from decreased levels of immune cells, such as CD4 + and CD8 + T lymphocytes. As a result, the reduced phagocytic capacity of macrophages leads to a pro-inflammatory state. This imbalance of immune cells is a common feature of aging, predisposing the elderly to various illnesses. In terms of COVID-19, a cytokine storm further exacerbates this imbalance, which may lead to acute myocarditis and eventual heart failure.

Both the incidence and the severity of COVID-19 infection increase with aging. Often, those highly vulnerable present with common comorbidities not limited to chronic heart disease, arrhythmias, cardiomyopathies, diabetes, coagulopathy, hypertension, and immuno-senescence. Followed by severe infection during the early phases of contracting the virus, patients may have a direct cardiac injury, increased inflammation, endothelialitis, and a cytokine storm (Costa et al., 2021).

Age is a significant prognostic indicator among COVID-19 patients. For a mean age group of 70 years, the main presenting symptoms include fever, shortness of breath, and a persistent cough. Elderly patients with cardiovascular comorbidities, particularly hypertension and diabetes, represent a significant group of critical cases with increased case fatality rates. With an upregulation of ACE2 receptors, the susceptibility to SARS-CoV-2 infection rises alongside their risk for acute COVID-19 cardiovascular syndrome (ACoVCS), cardiovascular disease, and an eventual MI. Up to 86% of elderly COVID-19 patients present with comorbidities, including chronic kidney disease (CKD), chronic obstructive pulmonary disease (COPD), congestive heart failure (CHF), hypertension (HTN), diabetes mellitus (DM), and cardiovascular disease (CVD). Most of these older adults presented with organ failure with secondary acute respiratory distress system (ARDS), cardiac injury, and/or liver damage, necessitating vasopressor support. Not only are elder patients with these comorbidities more likely to be infected, but they are also more likely to develop cardiovascular conditions and have worse outcomes secondary to the viral infection (Rowland and Kunadian, 2020).

In a retrospective analysis (Zhang et al., 2021) 562 patients hospitalized for COVID-19 at Wuhan Red Cross Hospital were divided into mild and severe illness criteria and assessed for their degree of underlying diseases. The severe group was significantly older averaging at 60.36 years, as compared to 40.23 years for the mild group. There was a statistically higher ratio of kidney impairment, liver damage, respiratory impairment, and coronary artery disease in the severe group. More patients in the severe group had pre-existing hypertension, diabetes, and malignancies unrelated to the viral infection. Laboratory studies indicated lower levels of lymphocytes, hemoglobin, and platelets in the severe group, while white blood cells, neutrophils, neutrophil to lymphocyte ratio, CRP, IL-6, procalcitonin, and erythrocyte sedimentation rate (ESR) were significantly elevated. Creatinine, troponin, creatine kinase, creatine kinase MB, and myoglobin were also significantly increased in the severe group. These laboratory results indicated that elderly patients were more likely to suffer from myocardial injury, vascular endothelial damage, and related hypercoagulation (Zhang et al., 2021). To explain this association, it is important to understand that most of the elderly patients in the severe group suffered from underlying conditions. These comorbidities progressed once they were infected with SARS-CoV-2, therefore heightening the critical nature of their condition. For this reason, it is essential to collect indications of a progressive illness and timely monitor the health of these high-risk patients to prevent deterioration of their conditions. These are not limited to assessing risk factors and warning signs of an immunocompromised state, as discussed next.

Early warning signs of a dysfunctional immune system include lymphopenia, increased levels of troponin, elevated BNP, and increased levels of CRP, IL-1β, IL-6 inflammatory markers. COVID-19 patients with an imbalance or elevation in these markers should be monitored as they are high-risk. If it is noted that the virus continues to proliferate, additional intervention treatments may be required. Particularly important to mention are elderly obese COVID-19 patients. This risk factor is itself linked with a more severe immune response to viral infection. The pro-inflammatory state in obese individuals is characterized by elevated inflammatory markers, as previously mentioned, leading to a stronger cytokine storm (Napoli et al., 2020).

As with many COVID-19 patients, a combination of risk factors plays a role in providing a prognostic indicator. Patients with pre-existing heart conditions may also be obese and suffering from comorbidities that warrant close monitoring. All patients with COVID-19 must be effectively assessed for comorbidities and associated risk factors. Appropriate testing, where and when appropriate, should be implemented to monitor cardiovascular functioning in elderly COVID-19 patients with severe illness. Not only does the level of care need to be the highest at the level of diagnosis, but also throughout the patient’s treatment regimen. To exercise optimal practices, emphasis must be placed on geriatric care, palliative care, advanced care planning, and management of comorbidities for the frailest, at-risk populations.

## Conclusion

COVID-19 infections present diverse effects on those with underlying heart conditions. The concern lies not only for patients with exacerbating comorbidities but also toward the onset and progression of new cardiovascular-related conditions. The cardiovascular manifestations associated with SARS-CoV-2 come in many different forms, however, what is clear is that the prognosis among patients is poor. Thus far, the knowledge of this novel virus is in its early stages. Nevertheless, with the current understanding of COVID-19, it is apparent that the cardiovascular system is intricately involved. The ability of the virus to cause electrolyte derangements causing cardiac arrhythmias, loss of ACE2 cardio-protection in MI, progression of hyperactive thrombosis via platelets, and age are only a few examples depicting the tip of the iceberg of this novel virus. For this reason, it is essential to explore and deduce the mechanisms by which SARS-CoV-2 operates to improve clinical outcomes for patients suffering from underlying cardiovascular diseases and reduce the risk of such conditions *de novo*.

## Author Contributions

GH, YB, and CK contributed to conception and design as well as wrote the first draft of the manuscript. JJ, NH, and MB wrote sections of the manuscript. All authors contributed to manuscript revisions, read, and approved the submitted version.

## Conflict of Interest

The authors declare that the research was conducted in the absence of any commercial or financial relationships that could be construed as a potential conflict of interest.

## Publisher’s Note

All claims expressed in this article are solely those of the authors and do not necessarily represent those of their affiliated organizations, or those of the publisher, the editors and the reviewers. Any product that may be evaluated in this article, or claim that may be made by its manufacturer, is not guaranteed or endorsed by the publisher.

## References

[B1] Ackermann M. Verleden S. E. Kuehnel M. Haverich A. Welte T. Laenger F. (2020). Pulmonary vascular endothelialitis, thrombosis, and angiogenesis in COVID-19. *N. Engl. J. Med.* 383 120–128. 10.1056/NEJMoa2015432 32437596 PMC7412750

[B2] Ashwin Reddy S. (2019). Ventricular arrhythmia precipitated by severe hypocalcaemia secondary to primary hypoparathyroidism. *Case Rep. Cardiol.* 2019:4851073. 10.1155/2019/4851073 31089428 PMC6476131

[B3] Atri D. Siddiqi H. K. Lang J. P. Nauffal V. Morrow D. A. Bohula E. A. (2020). COVID-19 for the cardiologist: basic virology, epidemiology, cardiac manifestations, and potential therapeutic strategies. *JACC Basic Transl. Sci.* 5 518–536. 10.1016/j.jacbts.2020.04.002 32292848 PMC7151394

[B4] Attwell D. Mishra A. Hall C. N. O’Farrell F. M. Dalkara T. (2016). What is a pericyte? *J. Cereb. Blood Flow Metab.* 36 451–455. 10.1177/0271678x15610340 26661200 PMC4759679

[B5] Baig A. M. Khaleeq A. Ali U. Syeda H. (2020). Evidence of the COVID-19 virus targeting the CNS: tissue distribution, host-virus interaction, and proposed neurotropic mechanisms. *ACS Chem. Neurosci.* 11 995–998. 10.1021/acschemneuro.0c00122 32167747

[B6] Bansal M. (2020). Cardiovascular disease and COVID-19. *Diabetes Metab. Syndr.* 14 247–250. 32247212 10.1016/j.dsx.2020.03.013 PMC7102662

[B7] Bhatla A. Mayer M. M. Adusumalli S. Hyman M. C. Oh E. Tierney A. (2020). COVID-19 and cardiac arrhythmias. *Heart Rhythm* 17 1439–1444. 32585191 10.1016/j.hrthm.2020.06.016 PMC7307518

[B8] Cardot-Leccia N. Hubiche T. Dellamonica J. Burel-Vandenbos F. Passeron T. (2020). Pericyte alteration sheds light on micro-vasculopathy in COVID-19 infection. *Intensive Care Med.* 46 1777–1778. 10.1007/s00134-020-06147-7 32533198 PMC7291173

[B9] Chen D. Li X. Song Q. Hu C. Su F. Dai J. (2020). Assessment of hypokalemia and clinical characteristics in patients with coronavirus disease 2019 in Wenzhou, China. *JAMA Netw. Open.* 3:e2011122. 10.1001/jamanetworkopen.2020.11122 32525548 PMC7290402

[B10] Chen T. Wu D. Chen H. Yan W. Yang D. Chen G. (2020). Clinical characteristics of 113 deceased patients with coronavirus disease 2019: retrospective study. *BMJ* 368:m1091. 10.1136/bmj.m1091 32217556 PMC7190011

[B11] Chen X. Zhao B. Qu Y. Chen Y. Xiong J. Feng Y. (2020). Detectable serum severe acute respiratory syndrome coronavirus 2 viral load (RNAemia) is closely correlated with drastically elevated interleukin 6 level in critically ill patients with coronavirus disease 2019. *Clin. Infect. Dis.* 71 1937–1942. 10.1093/cid/ciaa449 32301997 PMC7184354

[B12] Costa R. Castagna A. Ruotolo G. (2021). COVID-19 and cardiovascular problems in elderly patients: food for thought. *Aging Med.* 4 146–152. 10.1002/agm2.12149 33821229 PMC8014783

[B13] Darif D. Hammi I. Kihel A. El Idrissi Saik I. Guessous F. Akarid K. (2021). The pro-inflammatory cytokines in COVID-19 pathogenesis: what goes wrong? *Microb. Pathog.* 153:104799. 10.1016/j.micpath.2021.104799 33609650 PMC7889464

[B14] de Vries A. A. F. (2020). SARS-CoV-2/COVID-19: a primer for cardiologists. *Neth. Heart J.* 28 366–383. 10.1007/s12471-020-01475-1 32671650 PMC7360901

[B15] Dherange P. Lang J. Qian P. Oberfeld B. Sauer W. H. Koplan B. (2020). Arrhythmias and COVID-19: a review. *JACC Clin. Electrophysiol.* 6 1193–1204. 32972561 10.1016/j.jacep.2020.08.002 PMC7417167

[B16] Di Filippo L. Formenti A. M. Rovere-Querini P. Carlucci M. Conte C. Ciceri F. (2020). Hypocalcemia is highly prevalent and predicts hospitalization in patients with COVID-19. *Endocrine* 68 475–478. 10.1007/s12020-020-02383-5 32533508 PMC7292572

[B17] Drager L. F. Pio-Abreu A. Lopes R. D. Bortolotto L. A. (2020). Is hypertension a real risk factor for poor prognosis in the COVID-19 pandemic? *Curr. Hypertens. Rep.* 22:43. 10.1007/s11906-020-01057-x 32535705 PMC7292934

[B18] Driggin E. Madhavan M. V. Bikdeli B. Chuich T. Laracy J. Biondi-Zoccai G. (2020). Cardiovascular considerations for patients, health care workers, and health systems during the COVID-19 pandemic. *J. Am. Coll. Cardiol.* 75 2352–2371. 10.1016/j.jacc.2020.03.031 32201335 PMC7198856

[B19] Eltzschig H. K. Carmeliet P. (2011). Hypoxia and inflammation. *N. Engl. J. Med.* 364 656–665. 21323543 10.1056/NEJMra0910283 PMC3930928

[B20] Esler M. Esler D. (2020). Can angiotensin receptor-blocking drugs perhaps be harmful in the COVID-19 pandemic? *J. Hypertens.* 38 781–782. 32195824 10.1097/HJH.0000000000002450

[B21] Evans P. C. Rainger G. E. Mason J. C. Guzik T. J. Osto E. Stamataki Z. (2020). Endothelial dysfunction in COVID-19: a position paper of the ESC Working Group for atherosclerosis and vascular biology, and the ESC council of basic cardiovascular science. *Cardiovasc. Res.* 116 2177–2184. 10.1093/cvr/cvaa230 32750108 PMC7454368

[B22] Guan W. J. Ni Z. Y. Hu Y. Liang W. H. Ou C. Q. He J. X. (2020b). Clinical characteristics of coronavirus disease 2019 in China. *N. Engl. J. Med.* 382 1708–1720. 32109013 10.1056/NEJMoa2002032 PMC7092819

[B23] Guan W. J. Liang W. H. Zhao Y. Liang H. R. Chen Z. S. Li Y. M. (2020a). Comorbidity and its impact on 1590 patients with COVID-19 in China: a nationwide analysis. *Eur. Respir. J.* 55:2000547. 10.1183/13993003.00547-2020 32217650 PMC7098485

[B24] Guzik T. J. Mohiddin S. A. Dimarco A. Patel V. Savvatis K. Marelli-Berg F. M. (2020). COVID-19 and the cardiovascular system: implications for risk assessment, diagnosis, and treatment options. *Cardiovasc. Res.* 116 1666–1687. 10.1093/cvr/cvaa106 32352535 PMC7197627

[B25] He L. Mäe M. A. Muhl L. Sun Y. Pietilä R. Nahar K. (2020). Pericyte-specific vascular expression of SARS-CoV-2 receptor ACE2 – implications for microvascular inflammation and hypercoagulopathy in COVID-19. *bioRxiv* [Preprint]. 10.1101/2020.05.11.088500

[B26] Huang C. Wang Y. Li X. Ren L. Zhao J. Hu Y. (2020). Clinical features of patients infected with 2019 novel coronavirus in Wuhan, China. *Lancet* 395 497–506. 31986264 10.1016/S0140-6736(20)30183-5 PMC7159299

[B27] Jabri A. Kalra A. Kumar A. Alameh A. Adroja S. Bashir H. (2020). Incidence of stress cardiomyopathy during the coronavirus disease 2019 pandemic. *JAMA Netw. Open* 3:e2014780. 10.1001/jamanetworkopen.2020.14780 32644140 PMC7348683

[B28] Katzenstein A. L. Bloor C. M. Leibow A. A. (1976). Diffuse alveolar damage–the role of oxygen, shock, and related factors. A review. *Am. J. Pathol.* 85 209–228. 788524 PMC2032554

[B29] Klok F. A. Kruip M. van der Meer N. J. M. Arbous M. S. Gommers D. Kant K. M. (2020). Incidence of thrombotic complications in critically ill ICU patients with COVID-19. *Thromb. Res.* 191 145–147. 32291094 10.1016/j.thromres.2020.04.013 PMC7146714

[B30] Lau S. T. Yu W. C. Mok N. S. Tsui P. T. Tong W. L. Cheng S. W. (2005). Tachycardia amongst subjects recovering from severe acute respiratory syndrome (SARS). *Int. J. Cardiol.* 100 167–169. 10.1016/j.ijcard.2004.06.022 15820302 PMC7132412

[B31] Lazaridis C. Vlachogiannis N. I. Bakogiannis C. Spyridopoulos I. Stamatelopoulos K. Kanakakis I. (2020). Involvement of cardiovascular system as the critical point in coronavirus disease 2019 (COVID-19) prognosis and recovery. *Hellenic J. Cardiol.* 61 381–395. 10.1016/j.hjc.2020.05.004 32534109 PMC7286275

[B32] Lazzerini P. E. Boutjdir M. Capecchi P. L. (2020). COVID-19, arrhythmic risk, and inflammation: mind the gap! *Circulation* 142 7–9. 10.1161/CIRCULATIONAHA.120.047293 32286863

[B33] Lazzerini P. E. Capecchi P. L. Laghi-Pasini F. (2015). Long QT syndrome: an emerging role for inflammation and immunity. *Front. Cardiovasc. Med.* 2:26. 10.3389/fcvm.2015.00026 26798623 PMC4712633

[B34] Lazzerini P. E. Laghi-Pasini F. Boutjdir M. Capecchi P. L. (2019). Cardioimmunology of arrhythmias: the role of autoimmune and inflammatory cardiac channelopathies. *Nat. Rev. Immunol.* 19 63–64. 30552387 10.1038/s41577-018-0098-z

[B35] Li J. Wang X. Chen J. Zhang H. Deng A. (2020). Association of renin-angiotensin system inhibitors with severity or risk of death in patients with hypertension hospitalized for coronavirus disease 2019 (COVID-19) infection in Wuhan, China. *JAMA Cardiol.* 5 825–830. 10.1001/jamacardio.2020.1624 32324209 PMC7180726

[B36] Libby P. (2020). The heart in COVID-19: primary target or secondary bystander? *JACC Basic Transl. Sci.* 5 537–542. 10.1016/j.jacbts.2020.04.001 32292847 PMC7151324

[B37] Lippi G. South A. M. Henry B. M. (2020a). Electrolyte imbalances in patients with severe coronavirus disease 2019 (COVID-19). *Ann. Clin. Biochem.* 57 262–265. 10.1177/0004563220922255 32266828 PMC8173320

[B38] Lippi G. Wong J. Henry B. M. (2020b). Hypertension in patients with coronavirus disease 2019 (COVID-19): a pooled analysis. *Pol. Arch. Intern. Med.* 130 304–309. 10.20452/pamw.15272 32231171

[B39] Madjid M. Connolly A. T. Nabutovsky Y. Safavi-Naeini P. Razavi M. Miller C. C. (2019). Effect of high influenza activity on risk of ventricular arrhythmias requiring therapy in patients with implantable cardiac defibrillators and cardiac resynchronization therapy defibrillators. *Am. J. Cardiol.* 124 44–50. 10.1016/j.amjcard.2019.04.011 31047651

[B40] Mancia G. Rea F. Corrao G. (2020). RAAS inhibitors and risk of COVID-19. *N. Engl. J. Med.* 383 1990–1994. 10.1056/nejmc2030446 33108106

[B41] Mao L. Jin H. Wang M. Hu Y. Chen S. He Q. (2020). Neurologic manifestations of hospitalized patients with coronavirus disease 2019 in Wuhan, China. *JAMA Neurol.* 77 683–690. 10.1001/jamaneurol.2020.1127 32275288 PMC7149362

[B42] McCollum L. T. Gallagher P. E. Tallant E. A. (2012). Angiotensin-(1-7) abrogates mitogen-stimulated proliferation of cardiac fibroblasts. *Peptides* 34 380–388. 10.1016/j.peptides.2012.01.020 22326709 PMC3326596

[B43] Meng J. Xiao G. Zhang J. He X. Ou M. Bi J. (2020). Renin-angiotensin system inhibitors improve the clinical outcomes of COVID-19 patients with hypertension. *Emerg. Microbes Infect.* 9 757–760. 10.1080/22221751.2020.1746200 32228222 PMC7170368

[B44] Musher D. M. Abers M. S. Corrales-Medina V. F. (2019). Acute infection and myocardial infarction. *N. Engl. J. Med.* 380 171–176. 30625066 10.1056/NEJMra1808137

[B45] Napoli C. Tritto I. Benincasa G. Mansueto G. Ambrosio G. (2020). Cardiovascular involvement during COVID-19 and clinical implications in elderly patients. A review. *Ann. Med. Surg.* 57 236–243. 10.1016/j.amsu.2020.07.054 32802325 PMC7403130

[B46] Nguyen Dinh Cat A. Montezano A. C. Burger D. Touyz R. M. (2013). Angiotensin II, NADPH oxidase, and redox signaling in the vasculature. *Antioxid. Redox Signal.* 19 1110–1120. 10.1089/ars.2012.4641 22530599 PMC3771549

[B47] NIH (2020). *Study Suggests New Coronavirus May Remain on Surfaces for Days Office of Communications and Public Liaison in the NIH Office of the Director.* Available online at: https://www.nih.gov/news-events/nih-research-matters/study-suggests-new-coronavirus-may-remain-surfaces-days (March 24, 2020).

[B48] Patel V. B. Zhong J. C. Grant M. B. Oudit G. Y. (2016). Role of the ACE2/angiotensin 1-7 axis of the renin-angiotensin system in heart failure. *Circ. Res.* 118 1313–1326. 10.1161/CIRCRESAHA.116.307708 27081112 PMC4939482

[B49] Pena Silva R. A. Chu Y. Miller J. D. Mitchell I. J. Penninger J. M. Faraci F. M. (2012). Impact of ACE2 deficiency and oxidative stress on cerebrovascular function with aging. *Stroke* 43 3358–3363. 10.1161/strokeaha.112.667063 23160880 PMC3529166

[B50] Perico L. Benigni A. Casiraghi F. Ng L. F. P. Renia L. Remuzzi G. (2021). Immunity, endothelial injury and complement-induced coagulopathy in COVID-19. *Nat. Rev. Nephrol.* 17 46–64. 10.1038/s41581-020-00357-4 33077917 PMC7570423

[B51] Pillai P. Joseph J. P. Fadzillah N. H. M. Mahmod M. (2021). COVID-19 and major organ thromboembolism: manifestations in neurovascular and cardiovascular systems. *J. Stroke Cerebrovasc. Dis.* 30:105427. 10.1016/j.jstrokecerebrovasdis.2020.105427 33137615 PMC7584882

[B52] Pinto B. G. G. Oliveira A. E. R. Singh Y. Jimenez L. Goncalves A. N. A. Ogava R. L. T. (2020). ACE2 expression is increased in the lungs of patients with comorbidities associated with severe COVID-19. *J. Infect. Dis.* 222 556–563. 10.1093/infdis/jiaa332 32526012 PMC7377288

[B53] Porfidia A. Pola R. (2020). Venous thromboembolism in COVID-19 patients. *J. Thromb. Haemost.* 18 1516–1517. 10.1111/jth.14842 32294289 PMC7262050

[B54] Reynolds H. R. Adhikari S. Pulgarin C. Troxel A. B. Iturrate E. Johnson S. B. (2020). Renin-angiotensin-aldosterone system inhibitors and risk of COVID-19. *N. Engl. J. Med.* 382 2441–2448. 32356628 10.1056/NEJMoa2008975 PMC7206932

[B55] Rowland B. Kunadian V. (2020). Challenges in the management of older patients with acute coronary syndromes in the COVID-19 pandemic. *Heart* 106 1296–1301. 10.1136/heartjnl-2020-317011 32444504 PMC7253225

[B56] Roy D. Ghosh R. Dubey S. Dubey M. J. Benito-Leon J. Kanti Ray B. (2021). Neurological and neuropsychiatric impacts of COVID-19 pandemic. *Can. J. Neurol. Sci.* 48 9–24. 10.1017/cjn.2020.173 32753076 PMC7533477

[B57] Saad M. Omrani A. S. Baig K. Bahloul A. Elzein F. Matin M. A. (2014). Clinical aspects and outcomes of 70 patients with Middle East respiratory syndrome coronavirus infection: a single-center experience in Saudi Arabia. *Int. J. Infect. Dis.* 29 301–306. 10.1016/j.ijid.2014.09.003 25303830 PMC7110769

[B58] Segreto R. Deigin Y. (2021). The genetic structure of SARS-CoV-2 does not rule out a laboratory origin: SARS-COV-2 chimeric structure and furin cleavage site might be the result of genetic manipulation. *Bioessays* 43:e2000240. 10.1002/bies.202000240 33200842 PMC7744920

[B59] Setiadi A. Korim W. S. Elsaafien K. Yao S. T. (2018). The role of the blood-brain barrier in hypertension. *Exp. Physiol.* 103 337–342. 28986948 10.1113/EP086434

[B60] Shi S. Qin M. Shen B. Cai Y. Liu T. Yang F. (2020). Association of cardiac injury with mortality in hospitalized patients with COVID-19 in Wuhan, China. *JAMA Cardiol.* 5 802–810. 10.1001/jamacardio.2020.0950 32211816 PMC7097841

[B61] Siddiqi H. K. Libby P. Ridker P. M. (2021). COVID-19 – a vascular disease. *Trends Cardiovasc. Med.* 31 1–5. 33068723 10.1016/j.tcm.2020.10.005 PMC7556303

[B62] Skogestad J. Aronsen J. M. (2018). Hypokalemia-induced arrhythmias and heart failure: new insights and implications for therapy. *Front. Physiol.* 9:1500. 10.3389/fphys.2018.01500 30464746 PMC6234658

[B63] Solomon M. D. McNulty E. J. Rana J. S. Leong T. K. Lee C. Sung S. H. (2020). The COVID-19 pandemic and the incidence of acute myocardial infarction. *N. Engl. J. Med.* 383 691–693. 32427432 10.1056/NEJMc2015630

[B64] South A. M. Diz D. I. Chappell M. C. (2020). COVID-19, ACE2, and the cardiovascular consequences. *Am. J. Physiol. Heart Circ. Physiol.* 318 H1084–H1090. 10.1152/ajpheart.00217.2020 32228252 PMC7191628

[B65] Surawicz B. (1966). Role of electrolytes in etiology and management of cardiac arrhythmias. *Prog. Cardiovasc. Dis.* 8 364–386. 10.1016/s0033-0620(66)80011-7 5323242

[B66] Tadic M. Cuspidi C. Grassi G. Mancia G. (2020a). COVID-19 and arterial hypertension: hypothesis or evidence? *J. Clin. Hypertens.* 22 1120–1126. 10.1111/jch.13925 32627330 PMC7362072

[B67] Tadic M. Cuspidi C. Mancia G. Dell’Oro R. Grassi G. (2020b). COVID-19, hypertension and cardiovascular diseases: should we change the therapy? *Pharmacol. Res.* 158:104906. 10.1016/j.phrs.2020.104906 32461198 PMC7217779

[B68] Tang N. Bai H. Chen X. Gong J. Li D. Sun Z. (2020). Anticoagulant treatment is associated with decreased mortality in severe coronavirus disease 2019 patients with coagulopathy. *J. Thromb. Haemost.* 18 1094–1099. 32220112 10.1111/jth.14817 PMC9906401

[B69] Varga Z. Flammer A. J. Steiger P. Haberecker M. Andermatt R. Zinkernagel A. S. (2020). Endothelial cell infection and endotheliitis in COVID-19. *Lancet* 395 1417–1418. 10.1016/S0140-6736(20)30937-5 32325026 PMC7172722

[B70] Wang H. Pipes L. Nielsen R. (2021). Synonymous mutations and the molecular evolution of SARS-CoV-2 origins. *Virus Evol.* 7:veaa098. 10.1093/ve/veaa098 33500788 PMC7798566

[B71] Watkins J. (2020). Preventing a COVID-19 pandemic. *BMJ* 368:m810. 10.1136/bmj.m810 32111649

[B72] Whelton P. K. Carey R. M. Aronow W. S. Casey D. E. Jr. Collins K. J. Dennison Himmelfarb C. (2018). 2017 ACC/AHA/AAPA/ABC/ACPM/AGS/APhA/ASH/ASPC/NMA/PCNA guideline for the prevention, detection, evaluation, and management of high blood pressure in adults: a report of the American College of Cardiology/American Heart Association Task Force on Clinical Practice Guidelines. *Hypertension* 71 e13–e115. 29133356 10.1161/HYP.0000000000000065

[B73] Wrapp D. Wang N. Corbett K. S. Goldsmith J. A. Hsieh C. L. Abiona O. (2020). Cryo-EM structure of the 2019-nCoV spike in the prefusion conformation. *Science* 367 1260–1263. 32075877 10.1126/science.abb2507 PMC7164637

[B74] Xia S. Lan Q. Su S. Wang X. Xu W. Liu Z. (2020). The role of furin cleavage site in SARS-CoV-2 spike protein-mediated membrane fusion in the presence or absence of trypsin. *Signal Transduct. Target Ther.* 5:92. 10.1038/s41392-020-0184-0 32532959 PMC7289711

[B75] Xu J. Lazartigues E. (2020). Expression of ACE2 in human neurons supports the neuro-invasive potential of COVID-19 virus. *Cell. Mol. Neurobiol.* 1–5. 10.1007/s10571-020-00915-1 32623546 PMC7334623

[B76] Xu Z. Shi L. Wang Y. Zhang J. Huang L. Zhang C. (2020). Pathological findings of COVID-19 associated with acute respiratory distress syndrome. *Lancet Respir. Med.* 8 420–422. 10.1016/S2213-2600(20)30076-X 32085846 PMC7164771

[B77] Yarmohammadi H. Uy-Evanado A. Reinier K. Rusinaru C. Chugh H. Jui J. (2017). Serum calcium and risk of sudden cardiac arrest in the general population. *Mayo Clin. Proc.* 92 1479–1485. 28943016 10.1016/j.mayocp.2017.05.028 PMC5642050

[B78] Yu C. M. Wong R. S. Wu E. B. Kong S. L. Wong J. Yip G. W. (2006). Cardiovascular complications of severe acute respiratory syndrome. *Postgrad. Med. J.* 82 140–144. 10.1136/pgmj.2005.037515 16461478 PMC2596695

[B79] Zeng J. H. Wu W. B. Qu J. X. Wang Y. Dong C. F. Luo Y. F. (2020). Cardiac manifestations of COVID-19 in Shenzhen, China. *Infection* 48 861–870. 10.1007/s15010-020-01473-w 32725595 PMC7386384

[B80] Zhang J. Wang M. Ding W. Wan J. (2020). The interaction of RAAS inhibitors with COVID-19: current progress, perspective and future. *Life Sci.* 257:118142. 10.1016/j.lfs.2020.118142 32712300 PMC7377983

[B81] Zhang L. Peng Y. Zheng Q. Jiang L. Tang S. Chen P. (2021). Retrospective analysis of clinical characteristics and laboratory results of COVID-19 patients. *Eur. J. Inflamm.* 19:20587392211011919.

[B82] Zhang S. Liu Y. Wang X. Yang L. Li H. Wang Y. (2020). SARS-CoV-2 binds platelet ACE2 to enhance thrombosis in COVID-19. *J. Hematol. Oncol.* 13:120. 10.1186/s13045-020-00954-7 32887634 PMC7471641

[B83] Zhang Y. Moran A. E. (2017). Trends in the prevalence, awareness, treatment, and control of hypertension among young adults in the United States, 1999 to 2014. *Hypertension* 70 736–742. 10.1161/HYPERTENSIONAHA.117.09801 28847890 PMC5657525

[B84] Zheng J. Wong L. R. Li K. Verma A. K. Ortiz M. E. Wohlford-Lenane C. (2021). COVID-19 treatments and pathogenesis including anosmia in K18-hACE2 mice. *Nature* 589 603–607. 10.1038/s41586-020-2943-z 33166988 PMC7855185

[B85] Zhou F. Yu T. Du R. Fan G. Liu Y. Liu Z. (2020). Clinical course and risk factors for mortality of adult inpatients with COVID-19 in Wuhan, China: a retrospective cohort study. *Lancet* 395 1054–1062. 10.1016/s0140-6736(20)30566-3 32171076 PMC7270627

[B86] Zhu J. Ji P. Pang J. Zhong Z. Li H. He C. (2020). Clinical characteristics of 3062 COVID-19 patients: a meta-analysis. *J. Med. Virol.* 92 1902–1914. 10.1002/jmv.25884 32293716 PMC7262119

